# Competition is the basis of the transport mechanism of the NhaB Na^+^/H^+^ exchanger from *Klebsiella pneumoniae*

**DOI:** 10.1371/journal.pone.0182293

**Published:** 2017-07-27

**Authors:** Miyer Patiño-Ruiz, Constanța Ganea, Klaus Fendler, Octavian Călinescu

**Affiliations:** 1 Department of Biophysical Chemistry, Max Planck Institute of Biophysics, Frankfurt am Main, Germany; 2 Department of Biophysics, “Carol Davila” University of Medicine and Pharmacy, Bucharest, Romania; National Renewable Energy Laboratory, UNITED STATES

## Abstract

Na^+^/H^+^ exchange is essential for survival of all organisms, having a role in the regulation of the intracellular Na^+^ concentration, pH and cell volume. Furthermore, Na^+^/H^+^ exchangers were shown to be involved in the virulence of the bacterium *Yersinia pestis*, indicating they might be potential targets for novel antibiotic treatments. The model system for Na^+^/H^+^ exchangers is the NhaA transporter from *Escherichia coli*, EcNhaA. Therefore, the general transport mechanism of NhaA exchangers is currently well characterized. However, much less is known about NhaB exchangers, with only a limited number of studies available. The pathogen *Klebsiella pneumoniae*, which is a major source of nosocomial infection, possesses three electrogenic Na^+^/H^+^ exchangers, KpNhaA1, KpNhaA2 and KpNhaB, none of which have been previously investigated. Our aim in this study was to functionally characterize KpNhaB using solid supported membrane-based electrophysiology as the main investigation technique, and thus provide the first electrophysiological investigation of an NhaB Na^+^/H^+^ exchanger. We found that NhaB can be described by the same competition-based mechanism that was shown to be valid for electrogenic NhaA and NapA, and for electroneutral NhaP Na^+^/H^+^ exchangers. For comparison we also characterized the activity of KpNhaA1 and KpNhaA2 and found that the three exchangers have complementary activity profiles, which is likely a survival advantage *for K*. *pneumoniae* when faced with environments of different salinity and pH. This underlines their importance as potential antibiotic drug targets.

## Introduction

Na^+^/H^+^ exchangers are ubiquitous in nature as they ensure that organisms are capable of regulating their intracellular Na^+^ concentration, pH and volume [1]. The best studied Na^+^/H^+^ exchangers belong to the cation proton antiporter (CPA) superfamily, which includes both electrogenic and electroneutral members [2]. To this date, the crystal structures of four CPA Na^+^/H^+^ exchangers have been solved, beginning with the prototype of the family, NhaA from *E*. *coli* (EcNhaA) [3] and continuing in recent years with the structures of NapA from *T*. *thermophilus* [4], NhaP from *P*. *abyssi* [5] and NhaP1 from *M*. *jannaschii* [6]. We have previously shown that CPA Na^+^/H^+^ exchangers can be described by a simple kinetic model that explains their pH-dependent activity profile by competition of the two substrates, H^+^ and Na^+^, for a common binding site [7]. By comparison, less attention was given to the characterization of non-CPA Na^+^/H^+^ exchangers, which, despite having no homologues in humans, are present in prokaryotes and play roles in their Na^+^ or pH homeostasis. These include, but are not limited to, members of the NhaB [8], NhaC [9], or NhaD [10] families. Besides their value as model system for human homologues, investigation of prokaryotic Na^+^/H^+^ exchangers can serve two valuable roles–a better understanding of the general principles of the Na^+^/H^+^ exchange mechanism and, potentially, the development of novel antibacterial treatments, taking into account that the NhaA and NhaB exchangers have been shown to be involved in the virulence of *Y*. *pestis* [11]. In this respect, the absence of a NhaB homologue in humans may represent an advantage for the development of a side effect-free NhaB targeted antibiotic.

*Klebsiella pneumoniae* is facultative anaerobic rod-shaped bacterium belonging to the Enterobacteriaceae family that is ubiquitously found in nature, either in the environment or on the mucosal surfaces of mammals, including humans [12]. In humans, *Klebsiella* infects the respiratory and urinary tracts [12] and is a major source of hospital-acquired infections, threatening especially patients which are immuno-compromised and neonates in intensive care units [13]. A cause of major concern is the fact that, over the years, *K*. *pneumoniae* has acquired resistance to carbanepems, the first-choice drugs used in the treatment of *K*. *pneumoniae* infections [14]. The prevalence of carbanepem-resistant *K*. *pneumoniae* strains is continuously increasing, with one of the countries most severely affected being Greece, where in 2014, more than 60% of *K*. *pneumoniae* isolates from hospital wards were carbanepem-resistant [15]. A recently published systematic review of the literature points to a 42% pooled mortality rate of patients infected with carbanepem-resistant *K*. *pneumoniae*, double than that for those infected with carbanepem-susceptible strains [16]. Furthermore, it has been reported that *K*. *pneumoniae* strains have also started to develop resistance to last resort treatments such as colistin (polymyxin) [16]. In an effort to raise awareness regarding antibiotic-resistant bacteria, the World Health Organization has named carbanepem-resistant Enterobacteriaceae as Priority 1 pathogens against which antibiotic therapies have to be designed [17].

The genome of *K*. *pneumoniae* encodes for four Na^+^/H^+^ exchangers, two belonging to the NhaA family (KpNhaA1 and KpNhaA2), one belonging to the NhaB family (KpNhaB) and one belonging to the NhaP family (KpNhaP2). Out of these, according to the Transporter Classification system TC [18], three exchangers (KpNhaA1, KpNhaA2 and KpNhaP2) belong to the CPA superfamily, while KpNhaB belongs to the ion transporter (IT) superfamily. In terms of physiological role, NhaA and NhaB exchangers have been shown to mediate electrogenic Na^+^ (or Li^+^) export from the cell [19], having either a 2:1 H:Na^+^ stoichiometry (NhaA) [20] or a 3:2 H^+^:Na^+^ stoichiometry (NhaB) [21]. The role of the electroneutral NhaP exchangers is to regulate intracellular pH via import of Na^+^ ions [22]. No studies have so far been published regarding the Na^+^/H^+^ exchangers of *K*. *pneumoniae*.

**Fig 1:**
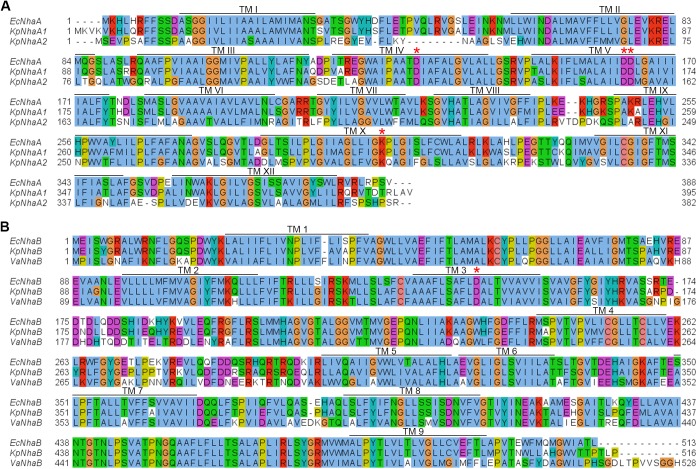
Sequence alignment of *K*. *pneumoniae* Na^+^/H^+^ exchangers. A, Sequence alignment of NhaA exchangers from *K*. *pneumoniae* (KpNhaA1, KpNhaA2) and *E*. *coli* (EcNhaA). B, Sequence alignment of NhaB exchangers from *K*. *pneumoniae* (KpNhaB), *E*. *coli* (EcNhaB) and *V*. *alginolyticus* (VaNhaB). Horizontal lines denote transmembrane helices in EcNhaA (A) or VaNhaB (B), as determined in [3] and [27], respectively. Red asterisks show conserved charged residues shown to be essential for transport function or stability of the exchangers. Alignments were performed using Clustal Omega [28] and drawn using Jalview [29].

A sequence alignment of the NhaA and NhaB exchangers investigated in this work with homologues from other species is shown in Fig 1. Extensive research on EcNhaA has revealed specific structural features and conserved residues that are critical to its function. Thus, TMs IV and XI (Fig 1A) are each interrupted by an unwound region [3], leading to the existence of short helices oriented either towards the cytoplasm (IVc, XIc) or towards the periplasm (IVp, XIp). The partial positive dipoles of helices IVc and XIp are stabilized by the presence of a negatively charged residue (Asp-133 in EcNhaA), which has been shown to be part of the substrate binding site [23] of EcNhaA. The partial negative dipoles of helices IVp and XIc are stabilized by Lys-300, which has recently been shown to be essential for the stability of the EcNhaA transporter [24]. Two neighbouring negatively charged residues, Asp-163 and Asp-164, are also part of the substrate binding site of EcNhaA [25], with mutations in these residues making the transporter inactive [26]. As it can be seen (Fig 1A), all these residues are conserved in the sequences of KpNhaA1 and KpNhaA2.

Unlike NhaA or NhaP transporters, which have 12 (or 13) transmembrane helices (TMs) and were shown to belong to the same structural fold termed the “NhaA fold” [30], NhaB was shown to have only 9 TMs [27]. Additionally, no clear homologues for the functionally relevant residues described for NhaA exist in the sequence of NhaB exchangers (Fig 1B). A mutational study done on the only negatively charged residue that seems to be present close to the middle of a TM in NhaB was performed in NhaB of *V*. *alginolyticus*, where mutation of Asp-147 to either Gly, Thr, Met or Glu abolished Na^+^/H^+^ exchange activity, but, interestingly, not Na^+^/Na^+^ exchange activity of the transporter [31].

The purpose of this work was the functional characterization of the KpNhaB Na^+^/H^+^ exchanger by using solid-supported membrane (SSM)-based electrophysiology [32] as the main investigation technique. Additionally, we also characterized KpNhaA1 and KpNhaA2 and compared their transport activities with that of KpNhaB. Our study represents the first electrophysiological investigation of an NhaB Na^+^/H^+^ exchanger. We found that the competition-based kinetic model [7] that is valid for CPA Na^+^/H^+^ exchangers also describes the non-CPA KpNhaB transporter. Additionally, we found that, despite their high homology to EcNhaA, KpNhaA1 and KpNhaA2 have altered activity profiles. Overall, the profiles of the three investigated Na^+^/H^+^ exchangers from *K*. *pneumoniae* are complementary, indicating that they contribute to the survival of the bacterium under different conditions of salinity and pH.

## Results

### Overexpression of KpNhaB, KpNhaA1 and KpNhaA2 in E. coli

**Fig 2:**
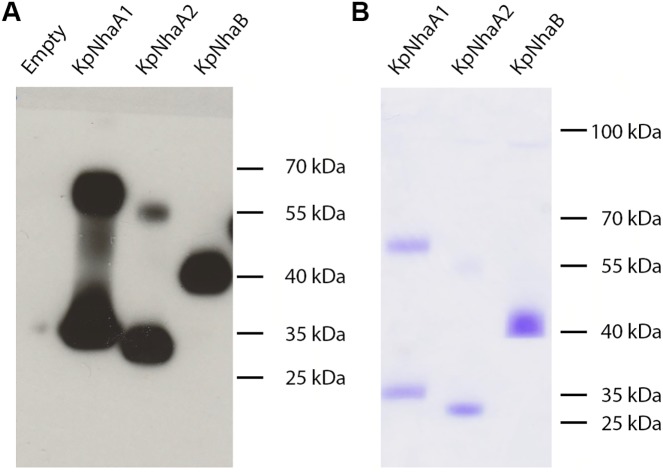
Expression in *E*. *coli* membranes and purification of *K*. *pneumoniae* Na^+^/H^+^ exchangers. A, *E*. *coli* membrane vesicles (100 μg total protein) were subjected to SDS-PAGE, followed by Western blot using an anti-His primary antibody. B, Purified KpNhaB, KpNhaA1 and KpNhaA2 (5 μg protein) were subjected to SDS-PAGE, followed by Coomassie Blue staining.

The genes encoding for KpNhaB, KpNhaA1 and KpNhaA2, containing a C-terminal His-tag and cloned in either the pET21d or the pTrcHis2 TOPO expression vectors, were used to transform *E*. *coli* strains BL21(DE3) or KNabc. Expression of the target proteins in *E*. *coli* membranes was verified by performing SDS-PAGE followed by Western blot, using anti-His IgG as primary antibody (Fig 2A). Clear bands could be observed for all three proteins on the Western blot. As NhaA was shown to be a dimer [33], we assigned the higher mass bands observed for KpNhaA1 and KpNhaA2 as the dimer forms of the protein. The existence of dimer bands even in the presence of the harsh detergent SDS has previously been reported for CPA Na^+^/H^+^ exchangers [34]. No dimer band was observed for KpNhaB. In all three cases, the monomeric forms of the proteins migrated on the gel to masses lower than their calculated molecular weights (KpNhaB– 57 kDa; KpNhaA1–43 kDa; KpNhaA2–41 kDa), which is typical for membrane proteins [35].

Following solubilization of the *E*. *coli* membranes and purification using immobilized metal affinity chromatography, proteins were subjected again to SDS-PAGE and visualized using Coomassie Blue staining of the gel (Fig 2B). Essentially only the same bands seen in Fig 2A were observed, indicating that proteins were purified to a high degree.

### Survival assays of E. coli KNabc expressing the K. pneumoniae transporters

In order to check the functionality of the expressed transporters in *E*. *coli* 7and also to verify their role as Na^+^ export systems, we assayed the capability of the *K*. *pneumoniae* exchangers of rescuing the survival of the Na^+^/H^+^ exchanger-deficient *E*. *coli* KNabc strain under conditions of high salinity. Under the conditions tested (Table 1), we observed that *E*. *coli* KNabc could only grow in medium where Na^+^ was replaced by K^+^ (LBK), while high concentrations of either Na^+^ or Li^+^ prevented the growth of the vector-transformed strain. When either the control NhaA from *H*. *pylori* (HpNhaA) or one of the *K*. *pneumoniae* Na^+^/H^+^ exchangers were expressed, survival of the cells was restored under high amounts of Li^+^ and Na^+^ at pH 7. However, at pH 8.3 only KpNhaA1 and HpNhaA could provide survival in presence of a high Li^+^ concentration, while none of the exchangers could provide survival under a high Na^+^ concentration.

**Table 1:** Survival of the Na^+^/H^+^ exchanger-deficient *E*. *coli* strain KNabc overexpressing *K*. *pneumoniae* Na^+^/H^+^ exchangers.

Exchanger	LBK	LBK pH 7 0.6 M NaCl	LBK pH 7 0.1 M LiCl	LBK pH 8.3 0.6 M NaCl	LBK pH 8.3 0.1 M LiCl
**KpNhaB**	++	+	++	–	–
**KpNhaA1**	+++	+++	+++	–	++
**KpNhaA2**	++	+	++	–	–
**HpNhaA**	+++	+++	+++	–	+++
**Vector**	+++	–	–	–	–

Survival was assessed under different salt concentration and pH values. Experiments were repeated at least three times with virtually identical results. NhaA from *H*. *pylori* (HpNhaA) was used as a positive control, while empty pTrcHis2TOPO vector was used as a negative control. +++ = maximum number of colonies, ++ = moderate number of colonies, + = small number of colonies,– = no growth detected. LBK = LB Broth containing K^+^ instead of Na^+^.

### Acridine orange dequenching assays

**Fig 3:**
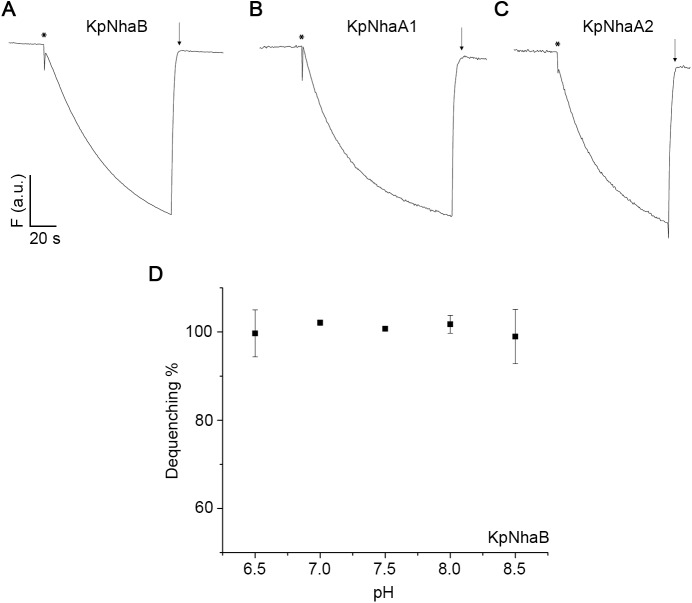
Acridine orange dequenching assays. *E*. *coli* KNabc membranes overexpressing KpNhaB (A), KpNhaA1 (B) and KpNhaA2 (C) were subjected to acridine orange dequenching assays at pH 8.5. The traces show the change of acridine orange fluorescence, F, over time. Addition of 2.5 mM Tris-D-lactate is marked by asterisks, while addition of 50 mM NaCl is marked by downward pointing arrows. D, pH dependence of the transport activity of KpNhaB recorded by acridine orange dequenching. Dequenching in D was induced by addition of 10 mM NaCl.

In order to ascertain the functionality of the overexpressed proteins in *E*. *coli* membranes, acridine orange dequenching assays were performed by using everted *E*. *coli* membrane vesicles that were acidified by addition of Tris-D-lactate. All three transporters were active (Fig 3A–3C), as shown by the dequenching of the acridine orange fluorescence observed when Na^+^ was added to the outside of the vesicles. As EcNhaB, which has high homology to KpNhaB (Fig 1B), was reported to show a pH-independent activity [36, 37], we recorded the pH dependence of the dequenching for KpNhaB and found essentially no pH dependence in the pH range tested (Fig 3D).

### SSM-based electrophysiological measurements

**Fig 4:**
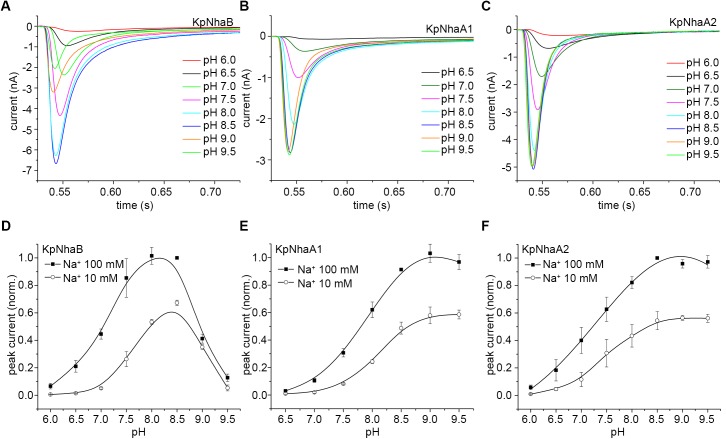
pH dependence of steady-state transport in *K*. *pneumoniae* Na^+^/H^+^ exchangers. A-C, Transient currents obtained following concentration jumps of 100 mM Na^+^ on KpNhaB (A), KpNhaA1 (B) and KpNhaA2 (C). D-F, Peak currents recorded following 100 mM or 10 mM Na^+^ concentration jumps on KpNhaB (D), KpNhaA1 (E) and KpNhaA2 (F). Data in D-F are presented as average of measurements performed on 3 different sensors ± s.d. and normalized to the maximum peak current. For (F), values for pH 9 and pH 9.5 were obtained following reconstruction of the transporter currents. Lines in D-F are guides to the eye.

Proteoliposomes containing the reconstituted *K*. *pneumoniae* Na^+^/H^+^ exchangers were investigated via SSM-based electrophysiology. Thus, the proteoliposomes were subjected to Na^+^ concentration jumps under conditions where the pH was the same inside and outside the proteoliposomes (symmetrical pH). In the case of all exchangers, transient currents of negative polarity were recorded (Fig 4A–4C). The negative polarity of these currents indicates the transport of positive charge out of the proteoliposomes (or net negative charge inside), and is in line with the expected electrogenicity of the Na^+^/H^+^ exchange. Concentration jumps of Li^+^ (data not shown) were also performed, yielding transient currents of the same polarity and shape as the ones recorded for Na^+^ concentration jumps.

### pH dependence of the transport activity

As previously shown [38], the amplitude of the recorded transient currents is a good measure of the steady-state transport activity of the exchanger, in the case where the recorded transient currents show no pre steady-state component. This was true for nearly all of the recordings done on the *K*. *pneumoniae* Na^+^/H^+^ exchangers, with the exception of Na^+^ concentration jumps on KpNhaB performed at pH 9 and pH 9.5, where the transient currents showed a pre steady-state component (Fig 4A). As this component appears only at high pH, it is likely associated with the Na^+^ binding or translocation event in the transporter’s reaction cycle, as was previously seen in the EcNhaA G338S mutant [38] or in the NhaP exchangers from *M*. *jannaschii* [22] and *P*. *abyssi* [39]. For these recordings, the transport currents were numerically reconstructed [40] and the stationary component of the reconstructed currents was determined (S1 Fig).

The pH-dependent activity profile of KpNhaB determined by SSM-based electrophysiology is shown in Fig 4D. A high pH dependence of the transport activity was observed (Fig 4D), unlike the profile determined by acridine orange dequenching measurements (Fig 3D). The exchanger was down-regulated in the acidic, with the activity increasing with pH up to a maximum that is reached at pH 8–8.5, after which activity decreased rapidly, reaching almost zero at pH 9.5 (Fig 4D). By comparison, KpNhaA1 and KpNhaA2 (Fig 4E and 4F) showed the same down-regulation in the acidic, but the activity remained high even at higher pH.

### Na^+^ and Li^+^ dependence of the transport activity

**Fig 5:**
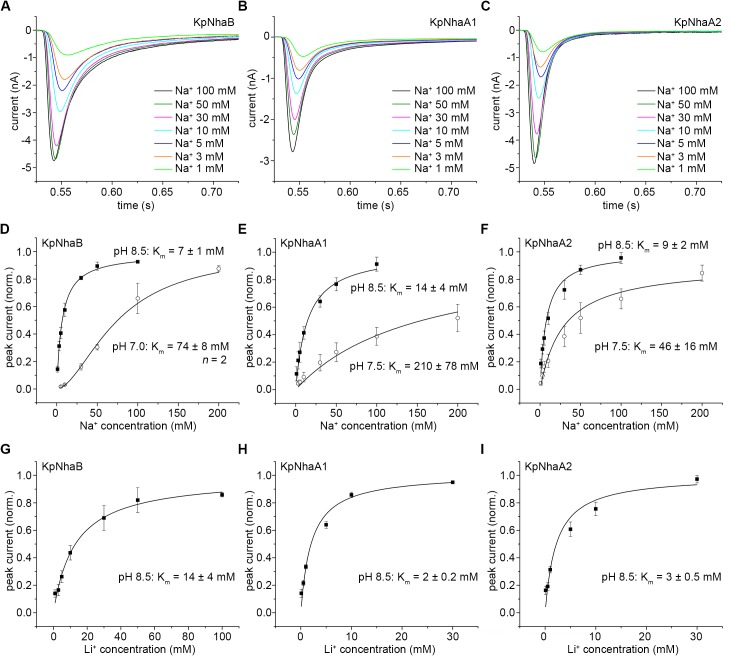
Substrate dependence of steady-state transport in *K*. *pneumoniae* Na^+^/H^+^ exchangers. A-C, Na^+^ dependence of transient currents following Na^+^ concentration jumps at pH 8.5 for KpNhaB (A), KpNhaA1 (B) and KpNhaA2 (C). D-F, Peak currents recorded following Na^+^ concentration jumps at various pH values for KpNhaB (D), KpNhaA1 (E) and KpNhaA2 (F). G-I, Li^+^ dependence of transport in KpNhaB (G), KpNhaA1 (H), KpNhaA2 (I) at pH 8.5. Curves represent hyperbolic fits to the data with the exception of the Na^+^ dependence of KpNhaB at pH 7 (D), where a Hill function was used. Data in D-I are normalized to the determined v_max_ values and presented as average of measurements performed on 3 independent sensors ± s.d.

At pH 8.5, increasing the value of the Na^+^ concentration jumps yielded a hyperbolic increase of the transporter-dependent currents, which was observed in all the investigated transporters (Fig 5A–5F). The determined values for the Na^+^ apparent affinities (K_m_ values) at pH 8.5 are similar for all three transporters, in the range of 7–14 mM Na^+^, and are also close to the value of 11 mM previously determined for EcNhaA at the same pH (Table 2).

**Table 2:** Kinetic parameters of *K*. *pneumoniae* Na^+^/H^+^ exchangers.

Exchanger	I_max_ (nA)	$K_{m}^{Na}\;(pH)$		$K_{m}^{Li}\;(pH)$	pK	$K_{D}^{Na}$	k_2_/k_1_	*n*	*m*
**KpNhaB**	6.1 ± 1	7 ± 1 (8.5)	74 ± 8 (7.0)	14 ± 4 (8.5)	8	3.6	23	1.3	1.6
**KpNhaA1**	3.3 ± 1	14 ± 4 (8.5)	210 ± 78 (7.5)	2 ± 0.2 (8.5)	9.2	1.6	13	1	1
**KpNhaA2**	4.3 ± 1	9 ± 2 (8.5)	46 ± 16 (7.5)	3 ± 0.5 (8.5)	8.4	2.6	100	1	1
EcNhaA^*^	12 ± 2	11 ± 1 (8.5)	102 ± 7 (7.5)	7 (8)	8.8	3	7	1	1

Values were determined following the fit of the model to the experimental data. Hill coefficients indicating cooperative Na^+^ (*n*) and H^+^ (*m*) binding were introduced for KpNhaB and fixed as 1 for the NhaA transporters which displayed no cooperative behavior. I_max_ indicates the maximum transient current amplitude recorded for the transporter.

^*^from Refs. [38, 41]. For EcNhaA, the I_max_ values were recorded for proteoliposomes reconstituted at a lipid to protein ratio of 5, while an LPR of 10 was used for the *K*. *pneumoniae* exchangers.

When pH was lowered, however, the peak currents recorded for KpNhaB at pH 7 showed a sigmoidal dependence (Fig 5D), indicating cooperativity in the substrate binding. In this case, the data could be fitted by the use of a Hill equation, yielding a Hill coefficient *n* = 2. The dependence of the currents on the Na^+^ concentration remained hyperbolical for KpNhaA1 (Fig 5E) and KpNhaA2 (Fig 5F). In all investigated exchangers, lowering pH had the effect of decreasing the affinity for Na^+^ (Fig 5 and Table 2), indicating the presence of competition between Na^+^ and H^+^.

We also performed Li^+^ concentration jumps at pH 8.5, given that most Na^+^/H^+^ exchangers are also capable of transporting Li^+^. KpNhaB displayed a twofold lower affinity for Li^+^ than for Na^+^ (Fig 5G). For KpNhaA1 (Fig 5H) and KpNhaA2 (Fig 5I), the affinities for Li^+^ were higher than the affinity for Na^+^, which is in line with the behavior previously seen in EcNhaA (Table 2).

### Determination of kinetic parameters

**Fig 6:**
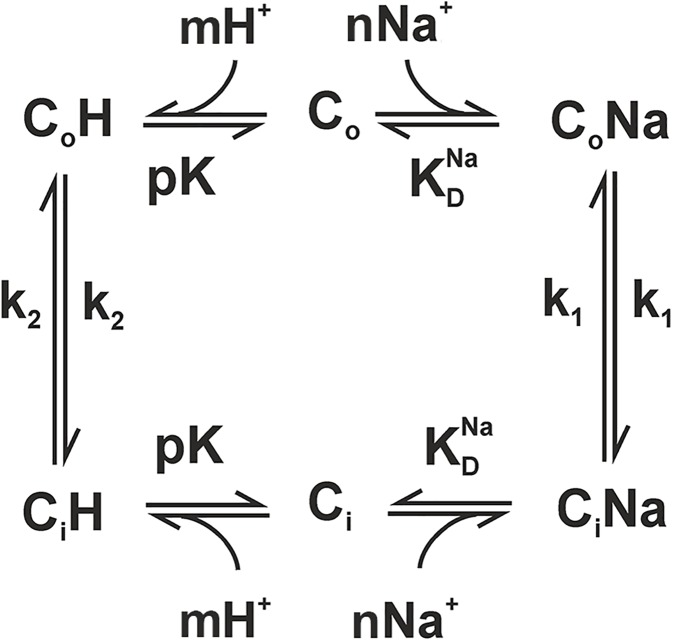
Kinetic model of Na^+^/H^+^ exchange. The exchanger can switch between an inside- (C_i_) or outside-open (C_o_) conformation only as long as one of the substrates (H^+^ or Na^+^) is bound. Binding of substrates is described by the constants pK and $K_{D}^{Na}$. Substrate translocation occurs with the rate constants k_1_ (for the translocation of Na^+^) or k_2_ (for the translocation of H^+^). *m* and *n* denote Hill coefficients for the binding of H^+^ and Na^+^, respectively.

Fig 6 shows the kinetic model describing the transport mechanism of Na^+^/H^+^ exchange, which is in accordance to the alternating access model proposed by Jardetzky [42]. In brief, the transporter can bind either of the substrates H^+^ and Na^+^ to the same binding site in either its inward- or outward-open conformations. This causes a conformational transition that exposes the bound substrate ion to the opposite side of the membrane, where it is released. One of the advantages of this simple model is that, in the absence of cooperative binding, the activity of each transporter can be described by a set of only 3 kinetic parameters (Fig 6), corresponding to the affinity of the transporter for H^+^ (pK), the affinity for Na^+^ ($K_{D}^{Na}$) and the ratio between the rates of H^+^ and Na^+^ transport (k_2_/k_1_).

We previously used the competition-based kinetic model in order to characterize several CPA exchangers, including transporters from the NhaA and NhaP families [7]. The pH-dependent activity profile of KpNhaB (Fig 4D) as well as the existence of competition (Fig 5D) indicated that the model might also be appropriate for the characterization of this non-CPA exchanger. In order to account for the cooperativity observed for KpNhaB at pH 7 we included two additional kinetic parameters, which are the Hill coefficients *n* for the Na^+^ binding reaction and *m* for the H^+^ binding reaction, respectively (Fig 6).

**Fig 7:**
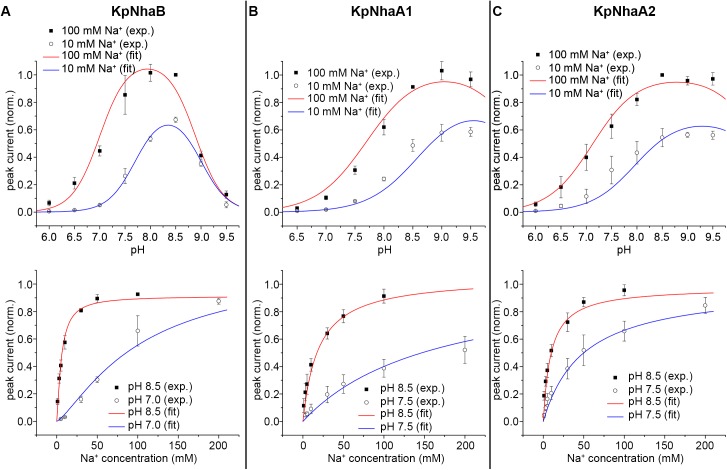
Fit of the kinetic model to the experimental data. The steady-state solution of the kinetic model was fitted simultaneously to the experimental data determined for the pH (top panel) and Na^+^ (bottom panel) dependences of KpNhaB (A), KpNhaA1 (B) and KpNhaA2 (C). Determined kinetic parameters are presented in Table 2. Exp. = experimentally determined data points, fit = modeled curve following the fit of the kinetic model to the experimental data.

A kinetic analysis of each of the investigated exchangers using the competition-based model was performed. Table 2 shows the obtained kinetic parameters for the investigated exchangers, and the fits of the model to the experimental data are shown in Fig 7. The *m* and *n* Hill coefficients were fixed as 1 for the NhaA exchangers, where no cooperativity was apparent.

As it can be seen (Table 2), the three transporters differ in their pK values, with KpNhaB being the most acid-shifted and KpNhaA1 the most alkaline-shifted. The determined $K_{D}^{Na}$ values are similar, in the range of 1.6–3.6 mM Na^+^.

Regarding k_2_/k_1_, a major aspect of obtaining a precise value is measuring the activity of the exchanger in a range where down-regulation at both acidic and alkaline pH can be observed. While this was the case for KpNhaB and KpNhaA1, where we obtained values for k_2_/k_1_ of 23 and 13, respectively, in the case of KpNhaA2 this value was less defined, most likely due to the weak down-regulation in the alkaline. Thus, although our fit gave a value of ~2000 to k_2_/k_1_ for KpNhaA2, we assigned it a k_2_/k_1_ value of 100, as values above 100 for this parameter yielded essentially identical pK and $K_{D}^{Na}$ values.

## Discussion

### KpNhaB, KpNhaA1 and KpNhaA2 are high turnover, electrogenic Na^+^ export systems

EcNhaA and EcNhaB have previously been shown to play a vital role in the survival of *E*. *coli* under conditions of high salinity [19]. Indeed, *E*. *coli* strains deficient in EcNhaA and EcNhaB such as the EP432 strain [43] or the KNabc strain that we employed in this work [44] do not survive under conditions of high Na^+^ or Li^+^ concentration.

As our results (Table 1) show, expressing either of the electrogenic *K*. *pneumoniae* Na^+^/H^+^ exchangers in *E*. *coli* KNabc restored the salt resistance of this strain at neutral pH. Conversely, at pH 8/0.6 M Na^+^, none of the expressed Na^+^/H^+^ exchangers, including the control HpNhaA, were able to restore resistance. This is in line with the higher susceptibility of *E*. *coli* KNabc to salt stress compared to other Na^+^/H^+^ exchanger-deficient strains such as EP432 [45, 46], and most likely results from the fact that, besides EcNhaA and EcNhaB, *E*. *coli* KNabc is missing also the unspecific ChaA transporter.

Thus, considering our results as well as the role of the NhaA and NhaB exchangers in other organisms [19, 45], we can conclude that the KpNhaB, KpNhaA1 and KpNhaA2 exchangers contribute to salt resistance in *K*. *pneumoniae*. This conclusion is additionally substantiated by the pH-dependent activity profiles of the exchangers (Fig 4D–4F), which are in line with the expected profile for an exchanger that has as a main role Na^+^ export [for an in-depth discussion, see Refs. 7 and 22].

An analysis of the transient currents recorded using SSM-based electrophysiology following Na^+^ (or Li^+^) concentration jumps clearly shows that all investigated exchangers are electrogenic, carrying net positive charge out of the proteoliposomes (or net negative charge inside) when Na^+^ is applied from the outside. This conclusion is substantiated by the negative polarity of the recorded transients, the decay time constants that are Na^+^ concentration dependent (Fig 5A–5C) and by the increase of the decay time constants of the transient currents with the increase of the lipid to protein ratio (LPR, S2 Fig), as previously shown for EcNhaA by Zuber *et al*. [41].

In addition, based on the maximum amplitude of the transient currents recorded for all *K*. *pneumoniae* Na^+^/H^+^ exchangers (Table 2), we can conclude that all of them are high turnover systems, as the recorded amplitudes are comparable with those previously recorded for EcNhaA (Table 2), which has a turnover of more than 1000 ions/second [47].

### KpNhaB can be described by the same competition-based mechanism as the NhaA exchangers

Our previous research activity has led to a simple competition-based transport mechanism (Fig 6) that we have shown to be valid for Na^+^/H^+^ exchangers of the CPA superfamily [7]. So far, the transporters that were shown to follow this mechanism were the NhaA exchangers from *E*. *coli* [38], *H*. *pylori* [22] and *S*. *typhimurium* [48], the NhaP exchangers from *M*. *jannaschii* [22] and *P*. *abyssi* [39] and the NapA exchanger from *T*. *thermophilus* [49]. The mechanism explains the highly pH-dependent activity of Na^+^/H^+^ exchangers as a consequence of its transport mechanism, not necessitating additional pH-sensitive regions (so-called “pH sensors”) other than the substrate binding site.

As we expected, the characterization of KpNhaA1 and KpNhaA2 was fully in line with the competition-based mechanism. Thus, competition is readily apparent in both exchangers as shown by the reduction of the Na^+^ affinity when pH decreases (Fig 5). Interestingly, we found that KpNhaB can equally be described by the competition mechanism. Thus, competition can be observed in KpNhaB by the same decrease in Na^+^ affinity with the lowering of pH as observed for the NhaA exchangers (Fig 5D). An intrinsic property of this mechanism (Fig 6) is that transport is down-regulated at extreme pH: in the acidic, down-regulation is explained by the H^+^ out-competing the Na^+^ ions, while in the alkaline, it is explained by H^+^ depletion that reduces the overall availability of this substrate and hence, turnover. Indeed, the pH profile of KpNhaB (Fig 4D) clearly shows both alkaline and acidic down-regulation while for KpNhaA1 (Fig 4E) and KpNhaA2 (Fig 4F) only acidic down-regulation is observed, most probably because alkaline down-regulation is shifted out of the experimentally accessible pH-range.

Our finding for KpNhaB is especially relevant taking into account that previous reports regarding the pH dependence of NhaB exchangers gave mixed conclusions [8, 36] and that NhaB belongs to a different transporter family than NhaA. In addition, NhaB has a distinct structure as far as conclusions from sequence analysis are predictive in this respect. We will treat these considerations in turn.

The first investigated NhaB exchanger was NhaB from *E*. *coli*, which was identified more than 25 years ago [50]. One of the earliest reported properties of EcNhaB was that its transport activity seemed independent of pH in the range 6.5 to 8.5, as seen in acridine orange dequenching assays [36, 37]. On the other hand, the activity of the NhaB exchanger from *V*. *alginolyticus* (VaNhaB) was shown to be highly pH dependent using the same assay [51]. The acridine orange dequenching assays performed on KpNhaB (Fig 3D) also showed no pH dependence of the transport activity in the same pH range. However, our electrophysiological results clearly show that the activity of KpNhaB is highly pH dependent (Fig 4D). Therefore, the apparent “pH insensitivity” observed in the acridine orange dequenching assay is most probably due to the limited dynamic range of the dequenching assays, as previously reported for *H*. *pylori* NhaA [52]. Here it is interesting to note that KpNhaB, just as *H*. *pylori* NhaA [52], has an acid-shifted activity profile with respect to EcNhaA. Using only the acridine orange dequenching assay, KpNhaB might, therefore, wrongly be qualified as “pH insensitive”, as HpNhaA was previously thought to be [52]. Thus, it is important that, when such considerations are made, a more sensitive assay than acridine orange dequenching is used.

According to the TC system [18], NhaB exchangers are classified into the Ion Transporter (IT) superfamily, while NhaA and NhaP exchangers belong to the Cation Proton Antiporter (CPA) superfamily. Furthermore, a topological study of VaNhaB showed that NhaB exchangers possess 9 transmembrane helices (TMs), unlike the 12 or 13 TMs present in NhaA or NhaP exchangers [27]. Also, NhaB exchangers, while electrogenic as NhaA exchangers, do not seem to possess obvious conserved motifs as were shown to be required for substrate transport in NhaA such as the two adjacent Asp residues present in TM V of NhaA (Fig 1A). However, a definite answer to the question how NhaA and NhaB transporters compare in terms of functional groups requires a high resolution structure which is presently not available.

Notwithstanding the structural dissimilarities between NhaA and NhaB type exchangers, we could establish that KpNhaB follows the same general mechanism as shown for the CPA exchangers. In particular, competition between H^+^ and Na^+^ obviously is a key element of the Na^+^/H^+^ exchange mechanism in these transporters and may be a general concept for all Na^+^/H^+^ exchangers.

The major difference between the behavior of KpNhaB and the NhaA exchangers was the observed cooperativity of Na^+^ binding in KpNhaB (Fig 5D), which was not found for the NhaA exchangers. This is a clear indication that more than one Na^+^ ion is involved in the transport mechanism and indeed this agrees with the different stoichiometries of Na^+^/H^+^ exchange: while NhaA transports 1 Na^+^ ion for 2 H^+^ [20], NhaB exchanges 2 Na^+^ ions for 3 H^+^ [21].

### Kinetic analysis of the investigated exchangers

The pH and Na^+^ dependence of all three investigated Na^+^/H^+^ exchangers from *Klebsiella pneumoniae* could be described by a simple kinetic model requiring only 3 (for NhaA type transporters) and 5 (for the NhaB type transporter) kinetic parameters (Fig 6). The fits of the model to the experimental data obtained for the three *K*. *pneumoniae* Na^+^/H^+^ exchangers are shown in Fig 7. The good quality of the fits and the low number of kinetic parameters represent strong support for the competition-based mechanism in all three transporters. The obtained pK and $K_{D}^{Na}$ values for the *K*. *pneumoniae* Na^+^/H^+^ exchangers (Table 2) are in line with previously determined kinetic parameters for Na^+^/H^+^ antiporters of other organisms. Thus, the Na^+^ affinity is virtually the same as that determined for the NhaA exchangers of *E*. *coli*, *H*. *pylori* and *S*. *typhimurium* [52]. The lowest pK value was determined for KpNhaB (8.0) and is the same as for *H*. *pylori* NhaA [52], while the highest was determined for KpNhaA1 (9.2) and is the same as for *S*. *typhimurium* NhaA [52].

### The role of *K*. *pneumoniae* Na^+^/H^+^ exchangers in bacterial survival

**Fig 8:**
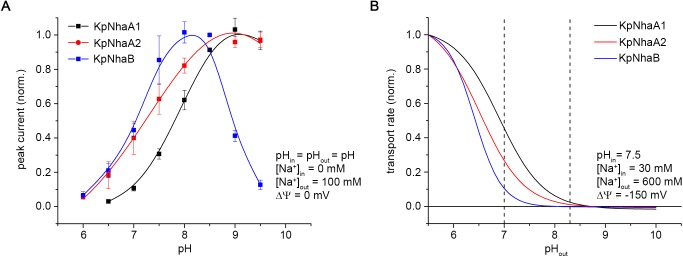
Activity profiles of *K*. *pneumoniae* Na^+^/H^+^ exchangers. A, Activity profile measured via SSM-based electrophysiology, under conditions of symmetrical pH (pH_in_ = pH_out_ = pH) and zero membrane potential (ΔΨ = 0). Data were taken from Fig 4. B, Activity profiles of *K*. *pneumoniae* Na^+^/H^+^ exchangers calculated using the determined kinetic parameters in Table 2 under physiological stress conditions and normalized to the transport rate determined at pH_out_ = 5.5. Parameters used are listed in the panel. Dashed lines in B denote pH 7 and 8.3, where the survival assays presented in Table 1 were performed. In A, *out* and *in* denote the exterior and interior of the proteoliposomes, respectively, while in B, *out* and *in* denote the periplasmic and cytoplasmic space, respectively.

The pH-dependent activity profiles of the three investigated exchangers obtained under symmetrical pH conditions are compared in Fig 8A, and can provide, along with the determined kinetic parameters, the basis of understanding the role of these exchangers in *K*. *pneumoniae*. However, it has to be kept in mind that a full comparison would require knowledge of the expression rate of the three exchangers, which we did not have access to.

In *E*. *coli*, EcNhaB is the main Na^+^ export system at acidic and neutral pH [19], while EcNhaA is highly up-regulated under conditions of salt stress [53] and is more efficient at removing Na^+^ at alkaline pH compared to EcNhaB [19]. Based on the kinetic parameters determined for the *K*. *pneumoniae* exchangers we modeled (Fig 8B) the behavior of these transporters under physiological stress conditions that *K*. *pneumoniae* can encounter, such as the ones that we used in our survival assays presented in Table 1. Unlike conditions used in the SSM experimental setup (Fig 8A), the physiological activity of the exchangers occurs under a negative-inside membrane potential and potentially at a different periplasmic pH and Na^+^ concentration compared to the cytoplasmic pH and Na^+^ concentration (Fig 8B).

It can be observed in Fig 8B that all three transporters are capable of exporting Na^+^ against a high concentration gradient at acidic pH. At high values of the salinity, KpNhaB is hard pressed to export Na^+^ once the periplasmic pH reaches neutral; under those conditions the two NhaA exchangers can perform this role, with KpNhaA1 being capable of functioning better at higher periplasmic pH. Overall, the three exchangers have complementary activity profiles (Fig 8B).

One more factor to consider is the behavior of the transporters when high amounts of Li^+^ are present. We have shown that KpNhaB has a comparatively lower affinity for Li^+^ (Table 2), whereas both KpNhaA1 and KpNhaA2 have much higher Li^+^ affinities (Table 2). Thus, it stands to reason that when the cell is faced with Li^+^ stress, the two NhaA exchangers are more adapted to ensure survival.

The modeled activity profiles can also be excellently correlated with the experimental results obtained in our survival assays (Table 1). Thus, at pH 7, in the presence of 600 mM Na^+^, all three exchangers are capable of ensuring survival of *E*. *col* KNabc, with the KpNhaA1-expressing cells surviving better. When pH is increased to 8.3, none of the expressed antiporters can restore survival of the strain in presence of 600 mM Na^+^, which fits very well the modeled activity in Fig 8B. An exception at pH 8.3 is represented by the capability of KpNhaA1 cells of surviving in 100 mM Li^+^, which can be explained by both the fact that this protein has the highest affinity for Li^+^ out of the exchangers investigated in this work and has also the most alkaline-shifted pK.

A better understanding of the way in which *K*. *pneumoniae* adapts to various stress conditions is essential, considering the danger posed by *K*. *pneumoniae* infections and the fact that many strains are resistant to currently used antibiotics. In all, the existence of three Na^+^/H^+^ exchangers with complementary transport profiles in *K*. *pneumoniae* indicates that this bacterium is well prepared to survive salt stress at various pH values. Designing specific, high affinity inhibitors against these Na^+^/H^+^ exchange systems is a possible avenue to consider in the task of finding a treatment against carbanepem-resistant *K*. *pneumoniae* infections.

## Materials and methods

### Genetic constructs and bacterial strains

The genes encoding the *K*. *pneumoniae* exchangers KpNhaA1 (Strain MGH 78578, Uniprot accession number: A6T4F6), KpNhaA2 (Strain MGH 78578, Uniprot accession number: A6TJ58) and KpNhaB (Strain 342, Uniprot accession number: B5XQ77) were synthesized by Genscript (Piscataway, NJ, USA) in the pET-21d(+) vector (Merck Millipore, Billerica, MA, USA). The genes were optimized for *E*. *coli* expression and contained an additional C-terminal 6-His tag. These constructs were used for protein production in the BL21(DE3) *E*. *coli* strain. For expression in the Na^+^/H^+^ exchanger deficient strain KNabc [44], the genes were cloned into the pTrcHis2 TOPO expression vector (Life technologies, Darmstadt, Germany) using the NcoI and EcoRI restriction sites. HpNhaA cloned in the pTrcHis2 TOPO expression vector obtained as previously described [22] was used as a control.

### Survival assays

Bacterial survival in presence of high concentrations of Na^+^ or Li^+^ was assessed essentially as previously described [23]. Briefly, *E*. *coli* KNabc cells, deficient in the expression of the Na^+^/H^+^ antiporters NhaA, NhaB and ChaA [44] were transformed with the KpNhaA1, KpNhaA2, KpNhaB, HpNhaA constructs or with the empty pTrcHis2 TOPO expression vector.

Cells were grown to OD_600_ of 0.6–0.7 in modified Luria-Bertani medium in which NaCl was replaced by KCl (LBK). The medium was buffered with 50 mM MOPS and contained 50 μg/ml kanamycin, 36 μg/ml chloramphenicol and 100 μg/ml ampicillin.

2 μl samples of serial 10-fold dilutions of the cultures were spotted onto LBK agar (1.5%) plates containing 0.6 M NaCl or 0.1 M LiCl at either pH 7 or 8.3 and incubated for 48 h at 37 °C. LBK agar plates with no addition of NaCl or LiCl were used as a control.

### Acridine orange dequenching in everted membrane vesicles

Everted vesicles from *E*. *coli* KNabc transformed with the recombinant plasmids for KpNhaA1, KpNhaA2 and KpNhaB were prepared as described previously [54]. Na^+^/H^+^ antiport activity was assessed based on the measurement of Na^+^-induced changes in the ΔpH as measured by acridine orange, a fluorescent probe of ΔpH. Everted vesicles were resuspended in buffer containing 10 mM Tris (titrated to the pH 7 using HCl), 250 mM sucrose and 140 mM choline chloride. Total protein concentration was measured using the Bradford assay [55].

Fluorescence was measured using a Hitachi F4500 Fluorimeter (Hitachi High-Technologies Corporation, Tokyo, Japan) at excitation and emission wavelengths of 495 nm and 530 nm, respectively. Dequenching assays were performed in buffer containing 10 mM MES (titrated to the indicated pH using Tris), 145 mM choline chloride, 5 mM MgCl_2_, 2 μM acridine orange. 100 μg (total protein) of everted vesicles were added to 1 ml external buffer. Acidification of the vesicles was induced using 2.5 mM Tris-D-lactate (at the corresponding pH). After reaching steady-state fluorescence, dequenching was induced by adding 10 or 50 mM NaCl. Finally, the pH gradient was dissipated by addition of 8 mM NH_4_Cl.

Dequenching was calculated as $Dequenching\;\%=\frac{F_{deq}-F_{min}}{F_{fin}-F_{min}}∙100$ where *F*_deq_ is the steady-state level of fluorescence achieved after dequenching, *F*_min_ is the steady-state level of fluorescence after Tris-D-lactate addition, and before Na^+^ addition, and *F*_fin_ is the steady-state level of fluorescence after dissipation of the pH gradient.

### Overexpression, purification and reconstitution

C-terminally His-tagged proteins were produced in *E*. *coli* BL21(DE3) cells and purified using immobilized Ni^2+^ affinity chromatography, as previously described [56]. Reconstitution of purified protein into proteoliposomes was performed using *E*. *coli polar* lipids extract (Avanti Polar Lipids, Alabaster, AL, USA) at a calculated LPR of 10 or 50, as previously described [38]. As previously shown [57], an LPR of 10 corresponts to a protein density of ~ 1000 protein particles/μm^2^.

### SSM-based electrophysiology

SSM measurements were performed as described previously [38]. Briefly, 30 μl of proteoliposomes at a lipid concentration of 3.3 mg/ml were adsorbed to an octadecanethiol / phospholipid hybrid bilayer on a gold surface (sensor). Unless otherwise stated, most measurements were performed using LPR 10 proteoliposomes. Proteoliposomes were allowed to adsorb to the sensor for 1 h. Electrogenic transport was initiated by a rapid change of substrate ion concentration in a single solution exchange protocol: non-activating solution (0.5 s)–activating solution (0.5 s)–non-activating solution (0.5 s). Currents were amplified using a current amplifier set to a gain of 10^8^−10^9^ V/A and a rise time of 10 ms.

Non-activating solutions contained 25 mM MES, 25 mM Hepes, 25 mM Tris, 5 mM MgCl_2_ and 300 mM KCl. Activating solutions contained 25 mM MES, 25 mM Hepes, 25 mM Tris, 5 mM MgCl_2_, x mM NaCl (or LiCl) and (300—x) mM KCl. All solutions were titrated to the desired pH using HCl or KOH.

In most cases, the amplitude of the recorded transient currents following Na^+^ concentration jumps was used in order to quantify steady-state Na^+^/H^+^ exchange activity. An exception was made for currents recorded for KpNhaB at pH 9.0 and pH 9.5, which showed a substantial pre steady-state component in addition to the steady-state component. In these cases, the currents were reconstructed according to the procedure described by Mager et al. [38] that allowed the measurement of the stationary component of the reconstructed current.

### Kinetic analysis

The steady-state solution for the kinetic model was calculated as described previously by Mager et al. [38]. This calculation yields turnover or activity of the transporter at a given Na^+^ concentration and pH. For KpNhaB, the model was modified by the addition of the Hill coefficients *m* and *n* that denote cooperative H^+^ and Na^+^ binding, respectively. Simultaneous fits of the model to the experimentally determined pH and Na^+^ dependences yielded values for the kinetic parameters pK, $K_{D}^{Na}$, k_2_/k_1_ and, in the case of KpNhaB, also for *m* and *n*. For the NhaA exchangers, where no cooperativity was apparent, *m* and *n* were fixed to 1.

## Supporting information

S1 Fig Measured *vs* reconstructed currents for KpNhaB. Current traces recorded for KpNhaB at pH 9.0 and 9.5 were reconstructed in order to determine the stationary component of the reconstructed current as described by Tadini-Buoninsegni and Fendler [40]. Presented is a trace recorded for a 100 mM Na^+^ concentration jump at pH 9.5, where there is a significant pre steady-state component.
(TIF)

S2 Fig Transient currents recorded for different values of the lipid to protein ratio (LPR). Current traces were recorded following 100 mM Na^+^ concentration jumps at pH 8.5 for KpNhaA1 (A), KpNhaA2 (B) and KpNhaB (C). For a better comparison of decay time constants at different LPR values, currents were normalized to their maximum amplitude.
(TIF)
