# Supplementary figures and images for: Competition is the basis of the transport mechanism of the NhaB Na+/H+ exchanger from Klebsiella pneumoniae
Source: PLoS One. 2017 Jul 27;12(7):e0182293. doi: 10.1371/journal.pone.0182293 (PMC5531510; doi:10.1371/journal.pone.0182293)

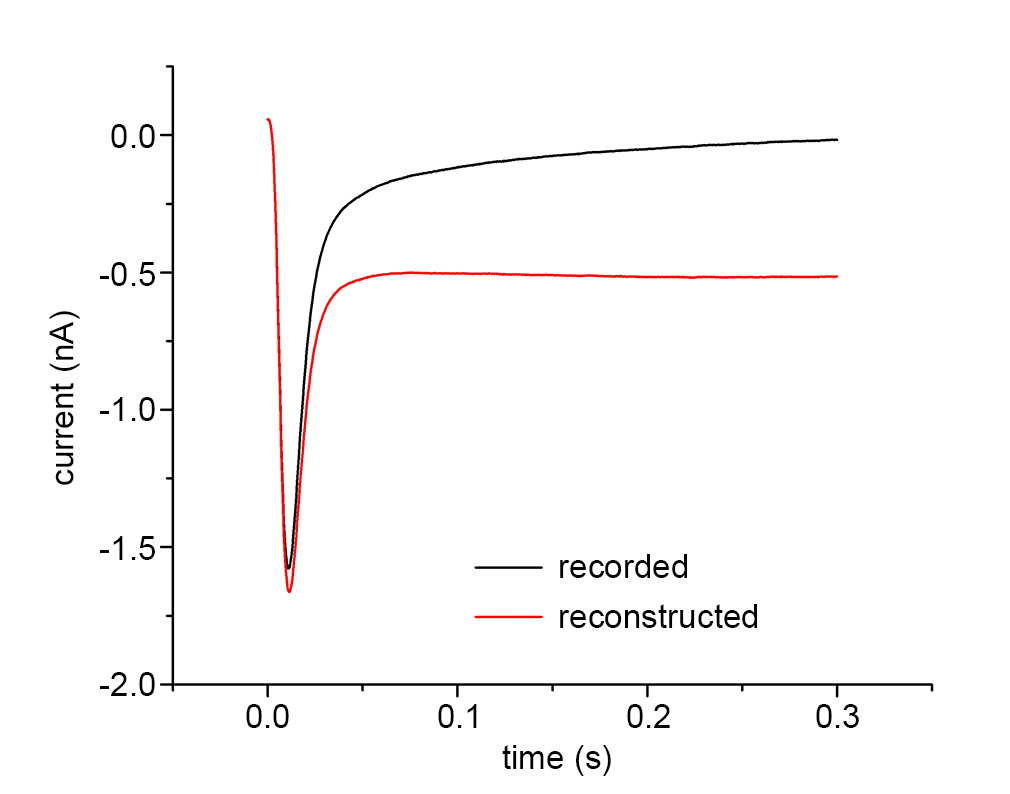

Supplement: S1 Fig — Current traces recorded for KpNhaB at pH 9.0 and 9.5 were reconstructed in order to determine the stationary component of the reconstructed current as described by Tadini-Buoninsegni and Fendler [40]. Presented is a trace recorded for a 100 mM Na+ concentration jump at pH 9.5, where there is a significant pre steady-state component. (TIF) [file pone.0182293.s001.tif]

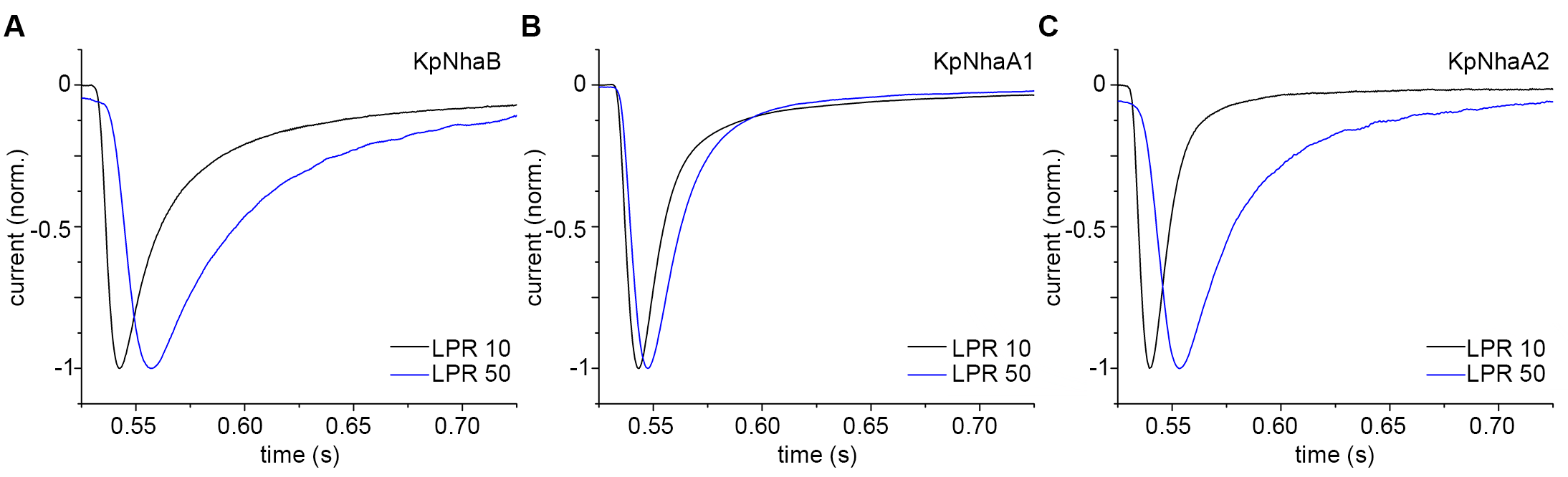

Supplement: S2 Fig — Current traces were recorded following 100 mM Na+ concentration jumps at pH 8.5 for KpNhaA1 (A), KpNhaA2 (B) and KpNhaB (C). For a better comparison of decay time constants at different LPR values, currents were normalized to their maximum amplitude. (TIF) [file pone.0182293.s002.tif]
